# Minocycline-Suppression of Early Peripheral Inflammation Reduces Hypoxia-Induced Neonatal Brain Injury

**DOI:** 10.3389/fnins.2017.00511

**Published:** 2017-09-12

**Authors:** Yingjun Min, Hongchun Li, Kaiyu Xu, Yilong Huang, Jie Xiao, Weizhou Wang, Longjun Li, Ting Yang, Lixuan Huang, Ling Yang, Hong Jiang, Qian Wang, Min Zhao, HaiRong Hua, Rong Mei, Fan Li

**Affiliations:** ^1^Department of Pathology and Pathophysiology, School of Basic Medical Science, Kunming Medical University Kunming, China; ^2^Department of Human Anatomy and Histoembryology, School of Basic Medical Science, Kunming Medical University Kunming, China; ^3^Department of Neurology, Yunnan First People's Hospital Kunming, China

**Keywords:** neonatal hypoxia, hypomyelination, leukocyte, minocycline, inflammation

## Abstract

While extensive studies report that neonatal hypoxia-ischemia (HI) induces long-term cognitive impairment via inflammatory responses in the brain, little is known about the role of early peripheral inflammation response in HI injury. Here we used a neonatal hypoxia rodent model by subjecting postnatal day 0 (P0d) rat pups to systemic hypoxia (3.5 h), a condition that is commonly seen in clinic neonates, Then, an initial dose of minocycline (45 mg/kg) was injected intraperitoneally (i.p.) 2 h after the hypoxia exposure ended, followed by half dosage (22.5 mg/kg) minocycline treatment for next 6 consecutive days daily. Saline was injected as vehicle control. To examine how early peripheral inflammation responded to hypoxia and whether this peripheral inflammation response was associated to cognitive deficits. We found that neonatal hypoxia significantly increased leukocytes not only in blood, but also increased the monocytes in central nervous system (CNS), indicated by presence of C-C chemokine receptor type 2 (CCR2^+^)/CD11b^+^CD45^+^ positive cells and CCR2 protein expression level. The early onset of peripheral inflammation response was followed by a late onset of brain inflammation that was demonstrated by level of cytokine IL-1β and ionized calcium binding adapter molecule 1(Iba-1; activated microglial cell marker). Interrupted blood-brain barrier (BBB), hypomyelination and learning and memory deficits were seen after hypoxia. Interestingly, the cognitive function was highly correlated with hypoxia-induced leukocyte response. Notably, administration of minocycline even after the onset of hypoxia significantly suppressed leukocyte-mediated inflammation as well as brain inflammation, demonstrating neuroprotection in systemic hypoxia-induced brain damage. Our data provided new insights that systemic hypoxia induces cognitive dysfunction, which involves the leukocyte-mediated peripheral inflammation response.

## Introduction

Neonatal encephalopathy due to perinatal asphyxia or hypoxia-ischemia (HI) is an important cause of mortality in the neonatal period and is associated with high morbidity, mainly characterized by neurological deficits such as, cognitive dysfunctions. While previous studies have associated focal ischemic injuries with hypoxic ischemic encephalopathy (HIE) (Vannucci and Hagberg, 2004; Salmaso et al., 2014), the most common injuries observed clinically are attributed to systemic hypoxia in pregnancy and/or delivery. Given that the immaturity of brain critically affected its susceptibility and underlying mechanism of HIE injury, especially at 24–32 weeks of human gestation (Back et al., 2001; Volpe et al., 2011; Salmaso et al., 2014), and that post-neonatal 0 day (P0d) rat is thought developmentally equivalent to a human pregnant 28 week fetal (Semple et al., 2013), in the present study, we used a systemic hypoxic model of P0d rat pups to investigate the unexplored long-term cognition alterations after the systemic hypoxic exposure at neonatal stage.

Cognitive dysfunction is the consequence of hypoxia-induced brain damage. Among the underlying mechanisms of hypoxia-induced neuronal injury, hypomyelination, and inflammation response have been commonly observed in the HIE model (Meng et al., 2006; Wang et al., 2008; Fatemi et al., 2011; Deng et al., 2014; Kaur et al., 2014). In addition, hypomyelination may be a consequence of inflammation after hypoxia exposure and it will result in long-term neuronal disorder (Volpe et al., 2011; Graf et al., 2014; Salmaso et al., 2014). Inflammation is a complex process consisting of an intrinsic network of multiple subsets of immune cells. Depending on injury setting, inflammation should include circulatory and brain endogenous local inflammatory responses (Anthony et al., 2012; Bonestroo et al., 2013). There are a number of studies demonstrating elevated microglia activation and IL-1β cytokine expression and release in the brain after hypoxia at the neonatal stage (Herrera-Marschitz et al., 2014; Kaur et al., 2014), and this brain endogenous inflammatory response is closely associated with brain damage and cognitive deficits (Fatemi et al., 2011; Alexandre et al., 2013) in HIE models. Notably, considerable research also revealed that peripheral immune cells such as, monocyte, macrophage, and neutrophil subtype of leukocytes infiltrated into CNS parenchyma in several adult rodent models including focal ischemia and experimental autoimmune encephalomyelitis, which contributed to inflammation/hypomyelination and neuronal injury (Morkos et al., 2007; Prinz and Priller, 2010; Herrera-Marschitz et al., 2014; Kichev et al., 2014). However, whether the peripheral leukocytes are associated with neonatal hypoxia-induced brain damage remains unknown. Given that the uncompleted development of the brain-blood-barrier (BBB) and the possible BBB broken down in response to hypoxia in neonates (Prinz and Priller, 2010), the peripheral leukocytes may invade into brain parenchyma and therefore play a critical role in inflammation/hypomyelination and cognitive dysfunction.

Minocycline, a tetracycline-antibiotic, has been not only reported to suppress microglial activation-mediated brain endogenous inflammation (Schmitz et al., 2014; Zhu et al., 2014), but also to reduce accumulation of IL-1β and Cytokine-induced neutrophil chemoattractants-1 (CINC-1) in the systemic circulation (Fox et al., 2005), as well as leukocyte transmigration and CNS infiltration (Brundula et al., 2002). Therefore, the neuroprotective role of minocycline might involve peripheral inflammation response. Extensive studies have suggested that minocycline is a neuroprotective agent that reduces brain injury in various animal models, including HI (Wixey et al., 2011a,b) and perinatal inflammation/infection hypoxia (Schmitz et al., 2014; Zhu et al., 2014). While the underline mechanisms are not fully understood, its anti-brain-inflammation effect seems critical. Therefore, in the present study, we aimed to examine how peripheral or brain residential inflammation responses to systemic hypoxia, and what roles they possible played in causing cognitive deficient and white matter hypomyelination.

## Materials and methods

### Animals

**Figure 1:**
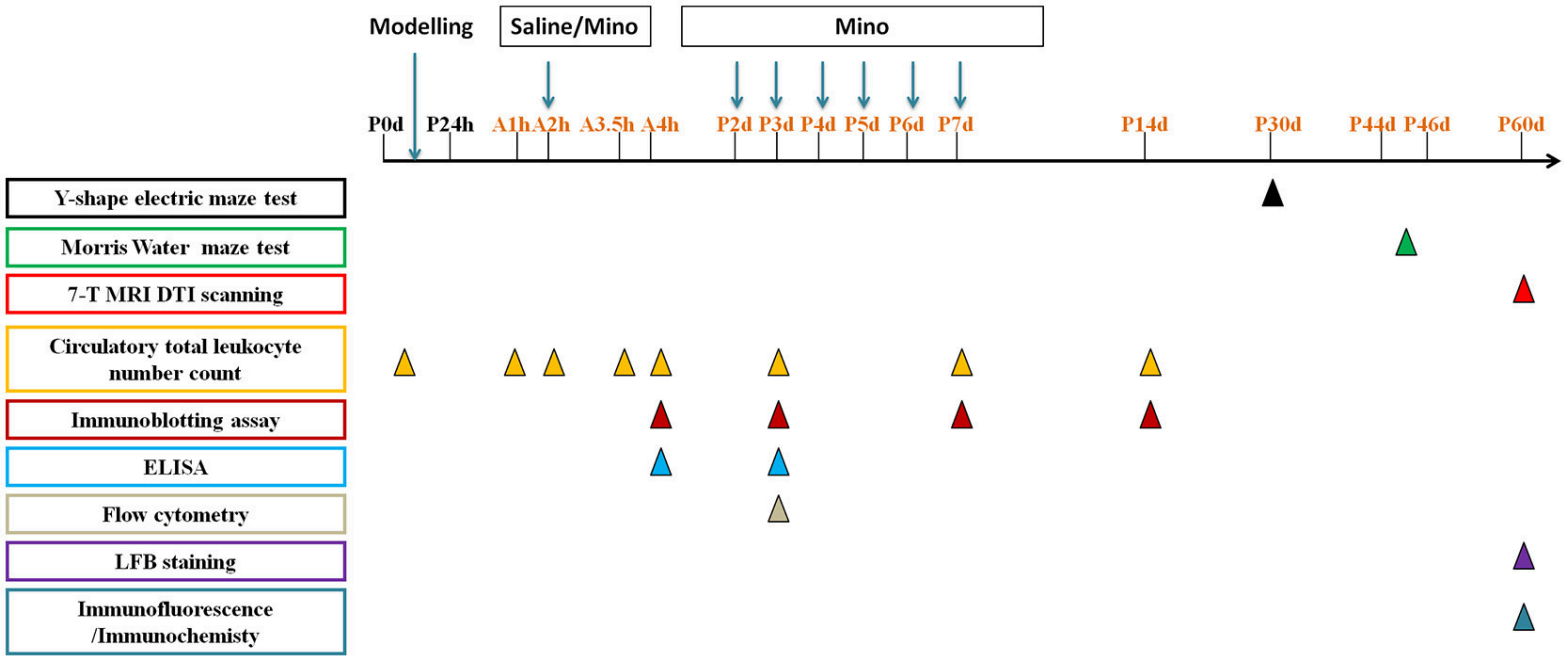
The time line of interventions in this study: 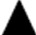 represents the time point to perform Y-shape electric maze test, 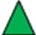 represents the time point to perform Morris Water maze test, 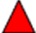 represents the time point to perform 7-T MRI DTI scanning, 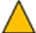 represents the time point to detect circulatory total leukocyte number count, 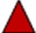 represents the time point to perform immunoblotting assay, 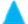 represents the time point to perform ELISA, 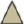 represents the time point to perform flow cytometry, 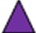 represents the time point to perform LFB staining, 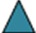 represents the time point to perform immunofluorescence/immunochemistry. Mino represents minocycline.

The animal care and experimental protocol (permit # SYXK2011-0004) was approved by the Animal Care and Use Committee of Kunming Medical University in accordance with the International Guiding Principles for Animals Research as stipulated by the Council for International Organizations of Medical Sciences (1985). Sprague Dawley (SD) breeders as well as their offspring were group-housed, with *ad libitum* access to water and food in the established animal houses, with a 12 h light/dark cycle and a thermoregulated environment. P0d rat pups (both sexes) were used for modeling the systemic hypoxia, and each experiment used 3–26 animals per group, the exact number as shown in figures and legends. The average mortality is 15%. And the time line of interventions was illustrated in Figure 1. All efforts were made to minimize the number of rats used and their suffering. The persons who performed animal behavior tests and brain imaging were blind to the treatment and group information.

### Systemic hypoxia and drug administration

P0d rat pups were kept in a hypoxia chamber (Chinese Utility Model Patent; patent number: ZL 2013 2 010770. 6) filled with nitrogen to remain 5% oxygen in the chamber at 28°C for 3.5 h or otherwise stated. Then the subjects were allowed to recover under normoxic condition for a certain of time as indicated. Some of the littermates were kept outside of the hypoxia chamber at 28°C at same time as normoxic matching controls. An initial dose of minocycline (45 mg/kg; Sigma-Aldrich, Louis, MO, USA) was injected intraperitoneally (i.p.) 2 h after the hypoxia exposure ended, followed by half dosage (22.5 mg/kg) minocycline treatment for six consecutive days daily (Vexler and Yenari, 2009). Saline was injected as vehicle control.

### Brain cell isolation

Neuronal cells were isolated from rat brain perfused with Ca^2+^/Mg^2+^ free Hanks' balanced salt solution. The cortex of the normal control group (NG), hypoxia-exposed group (Hy), and minocycline-injected hypoxia-insulted animals (Hy M) were dissected on ice, enzymatically digested using Neural Tissue Dissociation Kit (Miltenyi Biotec, Germany, cat:130-092-628), followed by myelin removal using myelin-conjugated magnetic beads (Miltenyi Biotec, Germany, cat:130-096-733) and LS column (Miltenyi Biotec, Germany,cat:130-042-401) as previously described (Li et al., 2015). Myelin-free cell fraction was centrifuged and the neuronal cell yield was collected for flow cytometry assay.

### Y-shape electric maze test

Y-shape electric maze (Chinese Utility Model Patent; patent number: ZL 2012 2 0666740. 5) behavior tests were performed at postnatal day 30 (P30d) to evaluate learning and memory. The Y-shape electric maze consisted of three arms (regions I–III), which converged to an equilateral triangular central area (region 0). Each arm has a 15 W lamp at the distal end and 50–70 volt electric stimulation was given on the floor in this apparatus. A safe arm was associated with illumination while the other arms and region 0 (dangerous arms) became regions with electrical foot stimulation. Rats were trained to learn and remember the illumination signal for escaping an electric shock. There were two steps in the test: Step 1: Adaption. Each rat was first placed at the end of one randomly chosen arm (starting arm) and was allowed to move freely in the maze during a 5 min session for adaptation. This session was repeated for 3 times. Step 2: Learning Training. The tested rats learned the illumination arm (safe arm) was safe so they would step into this arm to escape a shock. During this training period, the safe and stimulation arms were randomly chosen between trials. Twenty trails were performed daily with a 30 s intertrials interval. The latency that a rat escaped into the safe arm was recorded. We counted a trial as successful if the latency was shorter than 10 s. When a subject showed 90% of successful trials in a day, it was considered to have reached the learning criterion and the training was ceased. The number of days that animals took to reach the learning criteria (RLCD) was analyzed.

### Morris water maze test

After completion of the Y-maze behavior test, some of the animals (P44d–46d male) were subjected to Morris water maze testing to evaluate spatial reference learning and memory. The maze was a circular pool (180 cm in diameter) of water, maintained at 22–24°C, and divided into imaginary four quadrants. Objects were present around the water maze to be used as cues for spatial orientation. The behavior test was performed as previously described (Vorhees and Williams, 2006). Briefly, an opaque platform was placed in one of the quadrants during daily training. At the first day of adaptive practice (AP) training, a visible plastic platform (10 × 10 cm) was placed 0.5 cm above the surface of water, and the individual rat was allowed to swim and climb onto the platform within 120 s. Upon failure to locate the platform, the subject was guided to it and all individuals were allowed to rest on platform for 20 s and 10 min in hosting cage before next trial. Four trials with random start positions in the maze were given. This AP session was followed by 5 days of training sessions, in which the platform was hidden 1 cm below the surface of water. Rats were trained to locate the hidden platform during four trials per day as we described above. The time required for locating the submerged platform is termed “latency,” and the distance that animal traveled to find the platform is termed “swimming distance.” 24 h after the last training session (day 7), each animal was given a single 120 s probe test, in which the submerged platform was removed and the animal was expected to enter the target quadrant and make more entries into the location that previously contained the platform (memory retention and retrieval). Time spent in the target quadrant and the number of crosses in the platform location in the probe test were recorded by a video tracking system, and analyzed for evaluating the memory ability.

### 7-T MRI DTI scanning

A randomly chosen group of the animals (P60d male) after completion of the Morris water maze behavior test was subjected to MRI scanning. All MRI measurements were acquired utilizing a 7T Bruker scanner with a maximum gradient of 360 mT/m (Bruker BioSpec 70/20USR, Germany). A 200 mm birdcage transmit-only RF coil for both transmitting and an actively decoupled receive-only quadrature surface coil were used for adult brain MRI. Under inhaled isoflurane anesthesia (4% induction and 2% maintenance), animals were kept warm under circulating water at 37°C with continuous monitoring of the respiration rate. Whole-brain diffusion T2-weighted images (T2 DWI) were acquired with the following parameters: repetition time (TR), 3,000.0 ms; echo time (TE), 36.0 ms; resolution, 256 × 256; FA:180.0 deg, SI: 0.80/0.80 mm, FOV:3.50 cm^2^, MTX:256, Pos:0.40 mm 1:1, TurboRA- RE-T2 3:1. The diffusion T2-C-weighted images (T2-C DWI) were acquired with the following parameters: TR: 2,000.0 ms, TE: 36.0 ms, FA: 180.0 deg, SI: 0.80/0.80 mm, FOV: 3.50 cm^2^, MTX: 256, Pos: 2.00 mA 1:1, TurboRARE-T2-C 4:1. Diffusion tensor images (DTI) were acquired using an Echo Planar Imaging (EPI) sequence, TR: 6,000.0 ms, TE: 26.0 ms, FA: 90.0 deg, SI: 0.80/0.80 mm, FOV: 3.50 cm^2^, MTX: 128(a), Pos: 0.40 mmH 1:1, EPI-diffusion-tensor 5:1; 24 continuous slices between olfactory bulb and medulla oblongata were scanned. DTI images were analyzed using the Superconduction Magnet System Software. Briefly, fractional anisotropy (FA) maps of corpus callosum (CC) and middle corpus callosum (CCM) from 9/24 to 22/24 sections were averaged to generate a mean FA image, respectively. The person who performed this imaging was blind to the treatment and group information.

### Circulatory total leukocyte number count

As is described previously (Xu et al., 2013), the circulatory blood (60 μL) was collected from right ventricle 4 h, 3 days, 7 days, and 14 days after hypoxic exposure. Blood samples were put into a pre-anticoagulated tube and mixed well quickly. The leukocyte number was tested using the automatic blood cytoanalyzer (Sysmex XS-800i, Hyogo, Kobe, Japan).

### Flow cytometry

Single-cell myelin-free suspensions from different group was centrifuged and the pellet was resuspended in 100 μL blocking buffer containing CD32 (1:100, Biolegend, USA) for 10 min, followed by incubation in Fluorescence Activated Cell Sorter (FACS) staining buffer containing 2% Fetal Bovine Serum (BSA). Then, cells were incubated with antibody mixture at room temperature for 30 min, washed, centrifuged, resuspended in staining buffer and evaluated on BD C6 flow cytometer (BD Biosciences). The following combinations of antibodies with 1:100 dilution in staining buffer were used: anti-CD45-FITC(Biolegend)/CD11b/c-PE (Biolegend)/ CCR2-Alexa Fluor 647(Novus).

Compensation beads (BD Bioscience,USA) were incubated with antibody mixture (4°C, 30 min) and resuspended in staining buffer. Gating and data analysis were performed using FlowJo software (Tree Star, USA). The percentage of CCR2^+^/CD11b^+^CD45^+^ was calculated.

### Immunoblotting assay

The cerebral tissues containing the periventricular and hippocampus were lysed and proteins were extracted using a protein extraction kit (Biovision, Milpitas, CA, USA) containing phosphatase inhibitors (Roche, Penzberg, Germany) by following the manufacturer's protocol. Total protein concentration was determined using a commercial Bradford kit (Beyotime, Jiangsu, China). Equal amounts of protein 20 μg were loaded and separated by 10% sodium dodecyl sulfate-polyacrylamide gel (Beyotime, Jiangsu, China), and then transferred to polyvinylidene difluoride membrane (Millipore, Billerica, MA, USA) at 4 mA/cm^2^ of membrane for 70 min in mixture solution of Tris-Glycine buffer (TBS) and 20% (v/v) methanol. Membrane was blocked in TBS-Tween 20 0.2% (TBST) buffer containing 5% non-fat dry milk for 1 h at room temperature, and then incubated and gently shaken overnight at 4°C with primary antibody in TBST containing 5% non-fat dry milk; this was followed by incubation with corresponding secondary antibody for 2 h at room temperature. Rabbit anti-Iba-1 polyclonal IgG (1:1,500, Wako Chemicals, Richmond, VA, USA), rabbit anti-IL-1β polyclonal IgG (1:2,000, Abcam, Cambridge, UK), rabbit anti-TNF-α polyclonal IgG (1:1,500, Abcam, Cambridge, UK), rabbit anti-TGF-β1 polyclonal IgG (1:500,Santa Cruz, CA, USA), rabbit anti- CCR2 polyclonal IgG (1:1,000, Abcam, Cambridge, UK), mouse anti-GAPDH monoclonal IgG (1:30,000, Millipore, Billerica, MA, USA), and mouse anti-β-actin monoclonal IgG (1:20,000, Abcam, Cambridge, UK) were used as primary antibodies. The antibodies were detected using horseradish peroxidase conjugated anti-rabbit monoclonal IgG (1:3,000, Abmart, Shanghai, China) or anti-mouse monoclonal IgG (1:2,000–20,000, Abmart, Shanghai, China) secondary antibody and visualized using a chemiluminescence substrate system (Millipore, Billerica, MA, USA) on Chemiluminescence imagine system (Bio-Rad Laboratories, Hercules, CA, USA). Page Ruler prestained Protein Ladders (Thermo Fisher Scientific, Waltham, MA, USA) were used as molecular weight markers. Band optical density was quantified using Image J software (Rasband WS, Image J, US National Institutes of Health, Bethesda, MD, USA).

### Enzyme linked immunosorbent assay (ELISA)

At a series of time points (4 h and 3 d) after hypoxic exposure, pups were anesthetized with chloral hydrate (0.3 ml/100 g; ip) and blood samples were collected from the left ventricular of neonatal rat and stored at −4°C for 2 h to coagulate. Then, samples were centrifugalized for 20 min at 1,000 g and the supernatant was collected. Serum was aliquoted and frozen at −80°C before detection. The serum levels of interleukin 1 beta (IL-1β) and tumor necrosis factor alpha (TNF-α) were detected by Elisa kits (R&D Systems, cat# RLB00 and RTA00), were measured following the manufacturer's instructions, using an optical density reader (SPECTRAMAX190 reader, MD).

### The immunostaining of myelin basis protein (MBP)

Rats were anesthetized with chloral hydrate (0.3 ml/100g; ip) 60 days after hypoxia, followed by perfusion with PBS and then 4% paraformaldehyde. The brain was removed and post-fixed for 12 h at 4°C. Paraffin sections at 4 μm thickness were cut using a slicer (SLEE, CUT5062, Germany) and mounted onto gelatin-coated slides, then air-dried and stored at room temperature until use.

For MBP immunofluorescence staining, sections were de-waxed, rehydrated, and blocked with 5% donkey serum (Jackson Immuno Research, Cat: 017-000-121, USA) for 2 h at room temperature. Then rabbit anti-MBP antibody (1:100, abcam, Cat: ab40390, USA) was diluted in blocking solution and incubated at 4°C overnight. Subsequent antibody detection was carried out with Alexa Fluor 488 anti Rabbit IgG (1:200, invitrogen, Cat: A11010, USA) for 1 h. DAPI (1:1000, DOJINDO, Cat: D212, Japan) stained for 3 min before slides were coversliped with anti-quencher medium (VECTOR, Cat: H-1000, USA). A fluorescence microscope (Zeiss, Axio Observer Z1, Germany) was used to image our slides.

For MBP immunochemistry staining, sections were de-waxed, rehydrated, antigen retrieved (Khan et al., 2015), and rabbit anti-MBP antibody (1:100, abcam, Cat: ab40390, USA) was diluted in PBS solution and incubated at 37°C for 1 h. Followed by incubation with appropriate biotinylated secondary antibody (MXB, Cat:KIT-5010, China) for 15 min at 37°C. To visualize the immunoreactivity, sections were finally incubated with an avidin–biotin peroxidase complex solution (MXB, Cat:DAB-1031, China) for 3 min at room temperature. A light microscopic was used to exam the MBP expression.

### The luxol fast blue (LFB) staining

For LFB staining, brain sections were de-waxed and then incubated with Luxol Fast Blue staining solution (Solarbio,Cat:G3242,China) at 22–24°C for overnight. Then the sections were incubated in LFB differentiation solution (Solarbio,Cat:G1840, China) for 11 s, followed by rinse in 70% alcohol for 20 s. Sections were dehydrated in alcohol, cleared with xylene and then coversliped. A light microscope was used to exam the LFB staining.

### Statistical analysis

All data are presented as mean ± standard error of the mean (SEM). Normal distributed parameters were compared by two-tails Student's *t*-test for two comparisons and one-way analysis of variance (ANOVA) with Bonferroni *post-hoc* test for multiple comparisons. Mann-Whitney U test, Kruskal-Wallis test, and Friedman test were used to determine the statistical significance of distributed free parameters. The correlation analysis was performed by Spearman's rank correlation coefficient. Statistical analyses were performed with SPSS (IBM, Chicago, IL, USA). A value of *p* < 0.05 was considered statistically significant.

## Results

### Adverse effect of systemic hypoxia on learning and memory

**Figure 2:**
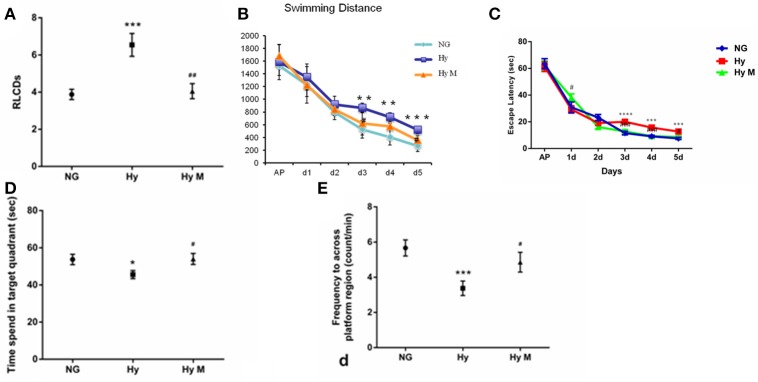
Learning and memory alterations after hypoxia with/without minocycline treatment. **(A)** In P30d rats, the average number of days that animals of indicated groups needed to reach RLCDs was obtained from Y-shape electronic maze test. ^***^p < 0.001 vs. the normal group (NG); ^##^*p* < 0.01 vs. the hypoxia group (Hy). *n* = 14–16 animals per group. **(B)** The swimming distance from day1 to day 6 in each group, ^**^p < 0.01 and ^***^p < 0.001 vs. NG group; ^#^*p* < 0.05 vs. Hy group. *n* = 16–20 animals per group. **(C)** Escape latencies in hidden-platform task during training in indicated groups. ^***^p < 0.001 and ^****^p < 0.0001 vs. NG group; ^#^*p* < 0.05 and ^###^*p* < 0.001 vs. Hy group. *n* = 16–20 animals per group. **(D,E)** In the probe test of the Morris water maze test, the time spent in the target quadrant **(D)** and the number of times crossing the platform region **(E)** of indicated groups were measured. ^*^p < 0.05 and ^***^p < 0.001 vs. NG group; ^#^*p* < 0.05 vs. Hy group. *n* = 14–16 animals per group. Data expressed as means ± SEM. NG represents normal control rats; Hy represents hypoxia rats; Hy M represents hypoxia rats with minocycline treatment.

We first evaluated whether the systemic hypoxia at P0d rat pups induced cognitive deficits. As shown in Figure 2A, when it was tested at P30d, animals with hypoxia (Hy) were trained for significantly more days to reach the learning critical days (RLCDs) in a Y-shape electric maze behavior task when compared to the control animals (NG; Figure 2A; *p* < 0.001 Hy vs. NG). Interestingly, Hypoxia-exposed rats with minocycline treatment (Hy M) significantly shortened the number of days to reach RLCDs during training (Figure 2A; *p* < 0.01 Hy M vs. Hy).

To further validate our findings observed in the Y-maze passive avoidance paradigm, we then performed Morris water maze spatial memory behavior tests in which animals must positively search for a hidden platform and remember its location. We found that hypoxia-exposed rats (Hy) had longer swimming distance (Figure 2B; *p* < 0.01 and *p* < 0.001 vs. NG group; *p* < 0.05 vs. Hy group) and longer latency to find the hidden platform compared to NG group (Figure 2C; *p* < 0.001 and *p* < 0.0001 Hy vs. NG), and this hypoxia-elongated latency and swimming distance were prevented by minocycline administration (Figures 2b,c; *p* < 0.05 and *p* < 0.001 Hy M vs. Hy). In the probe test, both the time spent in the target quadrant (Figure 2D) and the number of crossings in the platform region (Figure 2E) was significantly reduced in Hy P60d rats compared to that of the NG group (Figures 2D,E; *p* < 0.05 and *p* < 0.001 Hy vs. NG). Administration of minocycline after the onset of hypoxia for 7 days significantly increase the time spent in the target quadrant and the number of crossings in the platform region (Figures 2D,E; *p* < 0.05 Hy M vs. Hy). These data confirmed that systemic hypoxia at the neonatal stage induced a cognitive impairment, and the administration of minocycline is efficient to protect the brain against hypoxia even treated after the onset of the insult.

### Hypomyelination is associated with systemic hypoxia-induced brain injury

**Figure 3:**
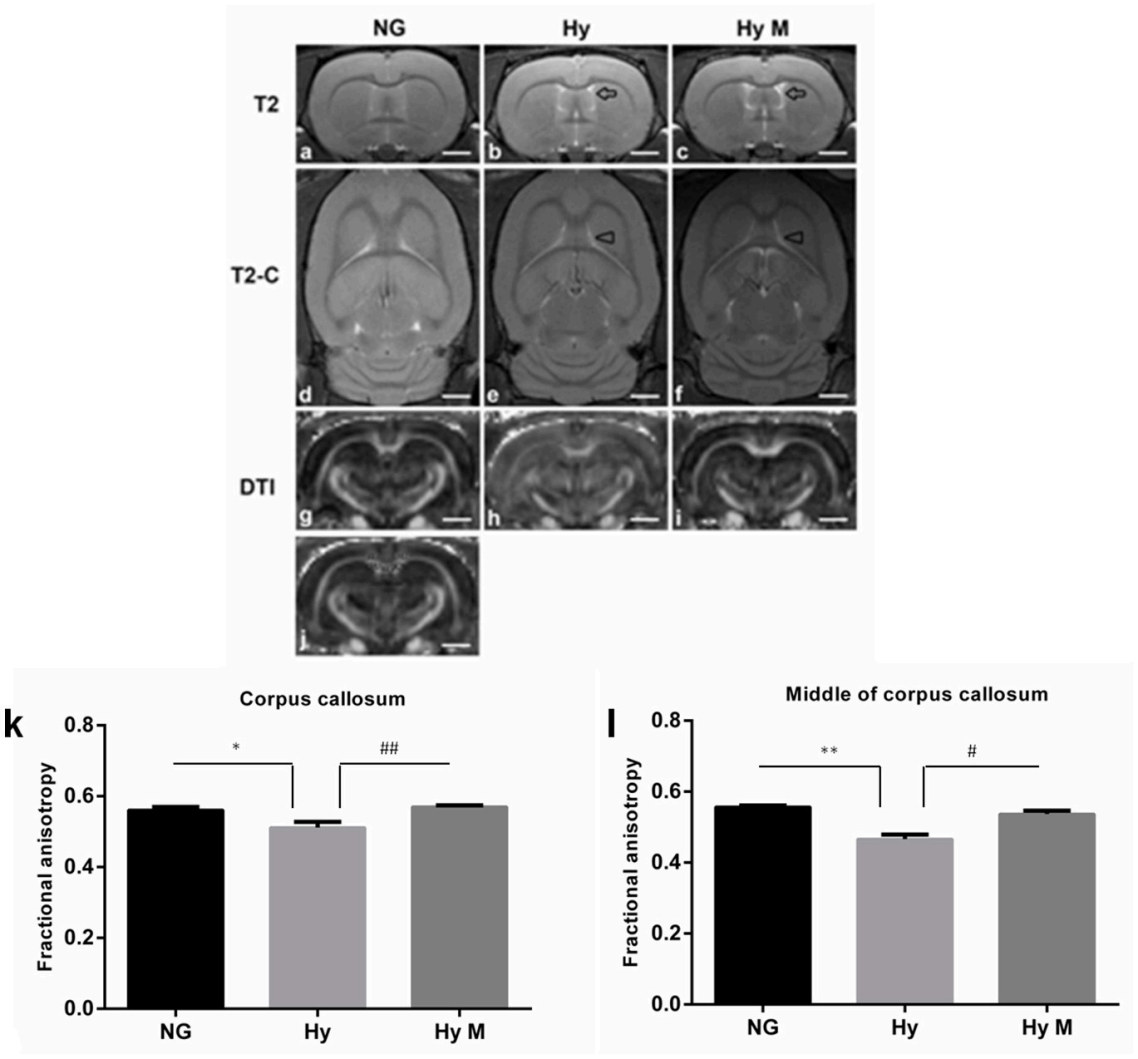
7T MRI/DTI images and the quantification of FA value in the targeted region of interest (ROI). **(a–i)** Representative T2, T2-C, and DTI images were obtained from the 13rd scanning section (with a clear CCM structure) of P60d rats in indicated groups. Arrows in T2 images indicated expanded septum and lateral ventricles. Arrowheads in T2-C images indicated the regions with reduced myelin sheath direction and destroyed myelin structure. **(j)** Illustration of the CC and CCM areas where the FA values were calculated. **(k,l)** The average of FA value of corpus callosum (CC) **(k)** and middle of CC **(l)** were measured and analyzed among indicated groups. ^*^p < 0.05 and ^**^p < 0.01 vs. NG group. ^#^*p* < 0.05 and ^##^*p* < 0.01 vs. Hy group. *n* = 5 animals per group. Data expressed as means ± SEM. NG represents normal control rats; Hy represents hypoxia rats; Hy M represents hypoxia rats with minocycline treatment. Scale bar = 2.5 mm.

As it is known that hypomyelination commonly happens in the HIE model (Meng et al., 2006; Wang et al., 2008; Fatemi et al., 2011; Deng et al., 2014; Kaur et al., 2014), here we tested if systemic hypoxia induced a hypomyelination, and investigated the effect of minocycline treatment after the onset of hypoxia on white matter protection. Compared to NG brain, expanded septum and lateral ventricles in Hy group were found in T2 images (Figures 3a,b) of the MRI scanning, reduced myelin sheath direction and destroyed myelin structure in the CC region was revealed in T2-C images (Figures 3d,e). These deleterious effects of hypoxia on white matter were attenuated by minocycline treatment (Figures 3c,f). In addition, the fractional anisotropy (FA) value that was detected by 7T MRI/DTI scanning (Figures 3g–j) was measured in the entire CC region and the CCM as well. When tested at P60d, a significant reduction of FA value was detected from both CC and CCM [region of interest (ROI) was illustrated in Figure 3j] of Hy group when compared to NG controls (Figures 3k,l; *p* < 0.05 in CC and *p* < 0.01 in CCM, respectively, NG vs. Hy), which demonstrated that persistent hypomyelination/white matter impairment existed in neonates after receiving systemic hypoxia. We also revealed that administration of minocycline restored the FA values in both CC and CCM regions (Figures 3k,l; *p* < 0.01 in CC and *p* < 0.05 in CCM, respectively, Hy M vs. Hy). Our data suggested that administration of minocycline is sufficient to protect white matter structure against hypoxia exposure in neonates.

**Figure 4:**
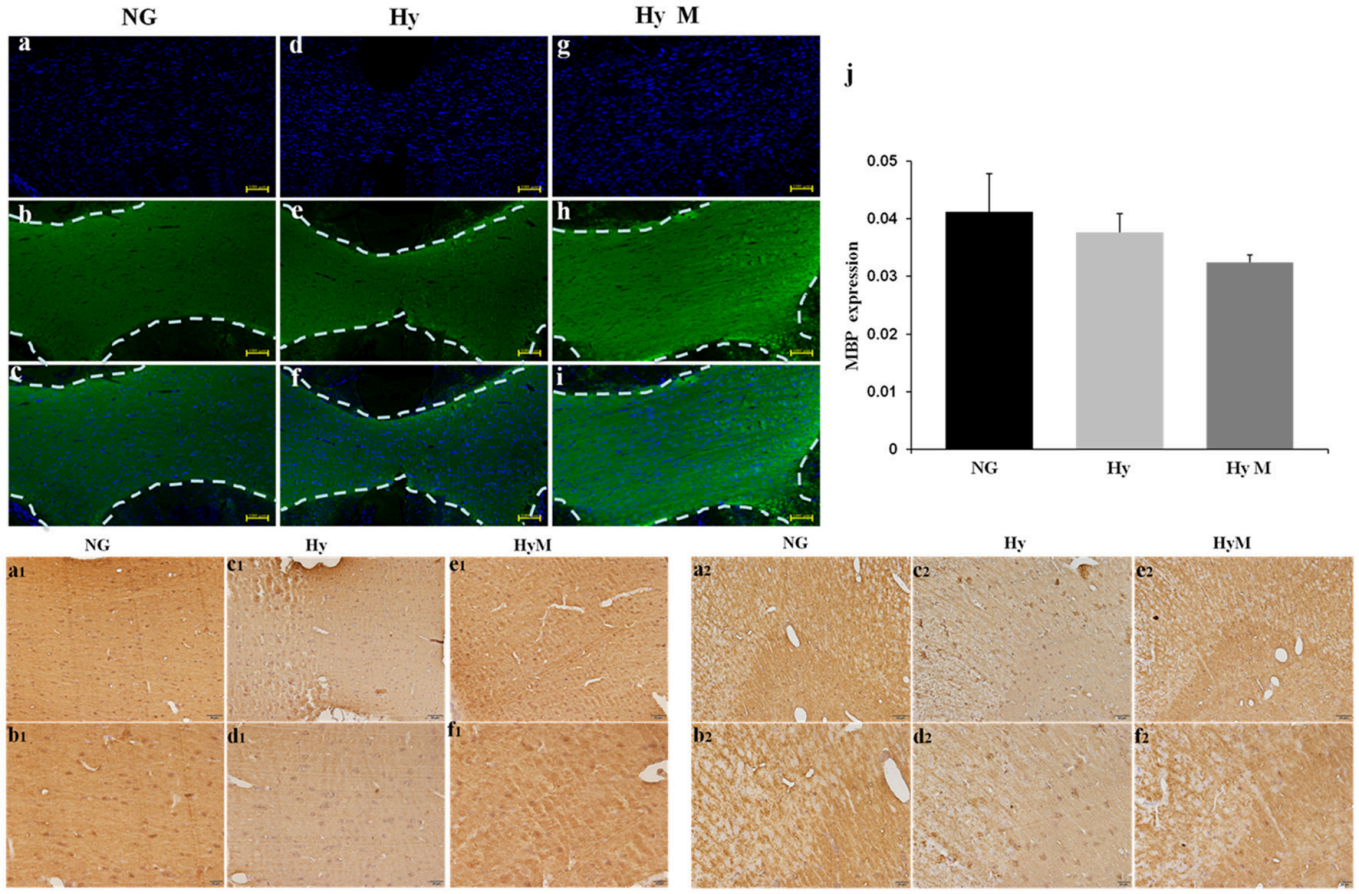
Immunostaining for detection of white matter changes in P60d rat brain after hypoxia. **(a–i)** The representative images of DAPI **(a,d,g)**, MBP immunofluorescence staining **(b,e,h)** and merged ones **(c,f,i)** in corpus callosum in indicated groups and **(j)** the quantification of MBP fluorescence intensity. Scale bar = 100 μm. *n* = 3 animals per group. Data expressed as means ± SEM. **(a1–f1)** The representative images of MBP immunohistochemistry staining in corpus callosum of indicated groups. Scale bar = 50 μm in the top panel and scale bar = 20 μm for the bottom panel. **(a2–f2)** The representative images of MBP immunohistochemistry staining in cingulum in indicated groups. Scale bar = 50 μm in the top panel and scale bar = 20 μm for the bottom panel. NG represents normal control rats, Hy represents hypoxia rats, Hy M represents hypoxia rats with minocycline treatment.

Next, immunochemistry staining was performed to exam the expression of myelin basis protein (MBP). As show in Figure 4, a thinner corpus callosum region (CC) was observed from hypoxia-exposed animal brain at 60 days after hypoxia (Figures 4a–j), although MBP protein expression level was not significantly altered (Figures 4a1–f1). Interestingly, MBP protein level was significantly decreased in cingulum area, but minocycline treatment ameliorated MBP decrease in cingulum region after hypoxia (Figures 4a2–f2).

**Figure 5:**
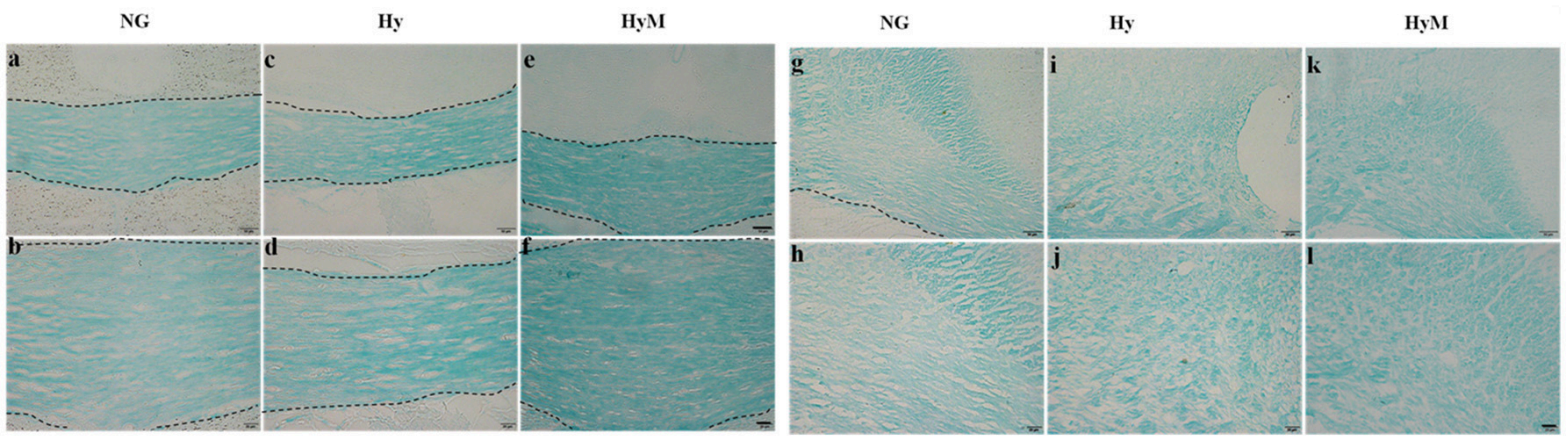
LFB staining for detection of white matter changes 60 days after hypoxia. The representative images of LFB staining in corpus callosum **(a–f)** and in cingulum **(g–l)** in indicated groups. Scale bar = 50 μm in the top and scale bar = 20 μm for the bottom. NG represents normal control rats, Hy represents hypoxia rats, Hy M represents hypoxia rats with minocycline treatment.

To reveal the structural changes of white matters in cingulum area, we performed histological LFB staining. We found a disordered myelin sheath arrangement in hypoxia-exposed animal brain. Not surprisingly, minocycline treatment ameliorated the myelin abnormalities at P60d animals after hypoxia (Figures 5a–l).

### Peripheral inflammation response is involved in neonatal systemic hypoxia

**Figure 6:**
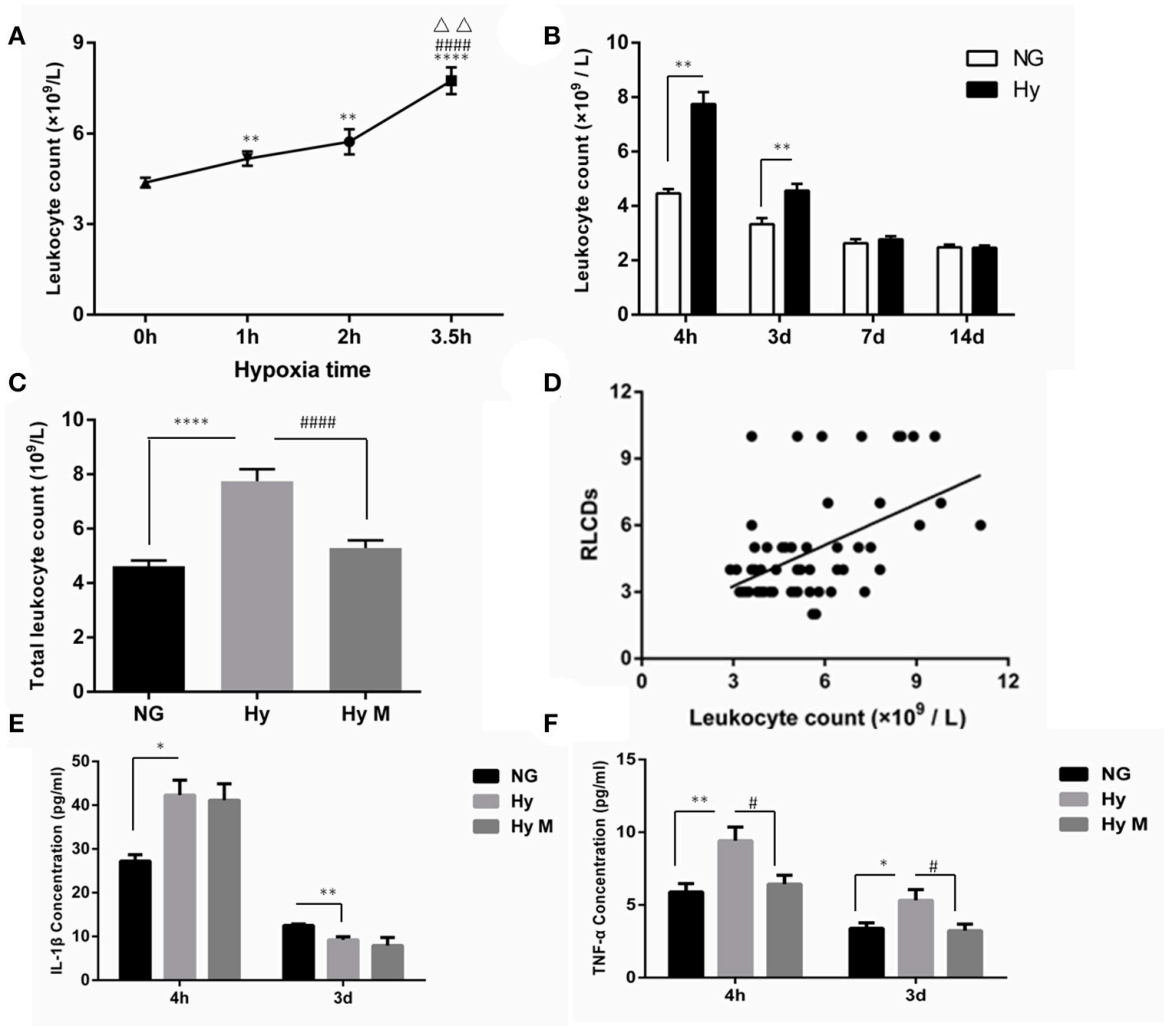
The circulatory leukocyte alteration, its relationship to cognitive dysfunction and inflammatory cytokines in peripheral blood after hypoxia. **(A)** The total number of leukocytes in blood was counted 4 h after hypoxia exposure for 1, 2, or 3.5 h. ^**^p < 0.01 and ^****^p < 0.0001 νs. 0 h group; ^####^*p* < 0.0001 vs. 1 h group; ^ΔΔ^*p* < 0.01 vs. 2.5 h group. *n* = 20–84 animals per group. **(B)** The total number of leukocytes in blood was determined at the indicated time points (4 h, 3 d, 7 d, 14 d) after 3.5 h hypoxia exposure. ^**^p < 0.01 vs. NG group. *n* = 26–30 animals per group. **(C)** Two hours after hypoxia (3.5 h), a single dose of minocycline (45 mg/kg) or vehicle was administered, and after another 2 h the blood samples were taken from indicated groups and the leukocytes were counted. ^****^p < 0.0001 vs. NG group. ^####^*p* < 0.0001 vs. Hy group. *n* = 25–26 animals per group. **(D)** The correlation between the total number of leukocytes accessed 4 h after hypoxia (3.5 h) with the behavior RLCDs score obtained 30 days after 3.5 h hypoxia exposure was analyzed, using the Spearman's rank correlation analysis. *n* = 20–22 animals per group. **(E,F)** ELISA was performed to determine the levels of IL-1β and TNF-α in serum at the indicated time points (4 h and 3 d) after 3.5 h hypoxia exposure. ^*^p < 0.05 and ^**^p < 0.01 vs. NG group. ^#^*p* < 0.05 vs. Hy group. *n* = 6–10 animals per group. Data expressed as means ± SEM. NG represents normal control rats; Hy represents hypoxia rats; Hy M represents hypoxia rats with minocycline treatment.

Given that hypomyelination may be a consequence of brain inflammation after hypoxia exposure (Volpe et al., 2011; Graf et al., 2014; Salmaso et al., 2014) and that inflammation consists of circulatory and brain endogenous inflammatory responses (Anthony et al., 2012; Bonestroo et al., 2013). We next investigated how the circulatory inflammation response, mainly indicated by leukocyte changes, responded to neonatal systemic hypoxia. We found that hypoxia exposure for 1 h significantly increased the total leukocyte number in blood when it was tested 4 h after hypoxia (Figure 6A; *p* < 0.01 1 h group vs. NG). A longer time of hypoxia exposure (2 h or 3.5 h) enhanced the leukocyte increase (Figure 6A; *p* < 0.01 and *p* < 0.0001, 2 h group and 3.5 h group vs. NG), and the statistical analysis identified that there is a significant correlation between time of hypoxia and the leukocyte number increase in blood (*r* = 0.600, *p* < 0.001). Then, we measured the leukocyte dynamics after hypoxia exposed for 3.5 h. As shown in Figure 6B, the total leukocyte number was significantly elevated by hypoxia initially 4 h after insult and then declined at 3 days after hypoxia (Figure 6B; *p* < 0.01 Hy vs. NG). The total number of leukocytes in blood remained normal when it was test 7 days or 14 days after hypoxia (Figure 6B; *p* > 0.05 Hy vs. NG).

To further evaluate the involvement of leukocytes in systemic hypoxia-induced brain damage, we performed the following two assessments. First, we investigated whether the minocycline would decrease the leukocyte number in the blood 4 h after hypoxia (3.5 h). As shown in Figure 6C, hypoxia-elevated leukocytes were significantly restored by minocycline administration (Figure 6C; *p* < 0.0001 Hy vs. Hy M). Second, a Spearman's rank correlation analysis indicated that the behavior scale of RLCDs was positively correlated with leukocyte number after hypoxia/minocycline treatment (Figure 6D; *r*_*s*_ = 0.501 and *p* < 0.0001).

In addition to the leukocytes response, we also measured the changes of inflammatory cytokines in serum. Four hours after the completion of neonatal hypoxia, serum IL-1β was significantly increased compared to normal group (Figure 6E; *p* < 0.05 Hy vs. NG). However, the concentration of serum IL-1β in hypoxia-exposed animals was significantly lower than control animal when it was detected 3 days after hypoxia exposure (Figure 6E; *p* < 0.01 Hy vs. NG). Treatment of minocycline had no effects on the neonatal hypoxia-induced serum IL-1β changes (Figure 6E; *p* > 0.05 Hy vs. Hy M). Another inflammatory cytokine, TNF-α, was elevated by neonatal hypoxia 4 h and 3 days after exposure (Figure 6F; *p* < 0.01 and *p* < 0.05 Hy vs. NG). Minocycline administration significantly suppressed TNF-α elevation after neonatal hypoxia (Figure 6F; *p* < 0.05 Hy vs. Hy M). These data suggested that leukocytes not only responded to hypoxia but also positively correlated with brain function alterations and minocycline may inhibit the peripheral inflammation response.

### Brain endogenous inflammatory response after systemic hypoxia

**Figure 7:**
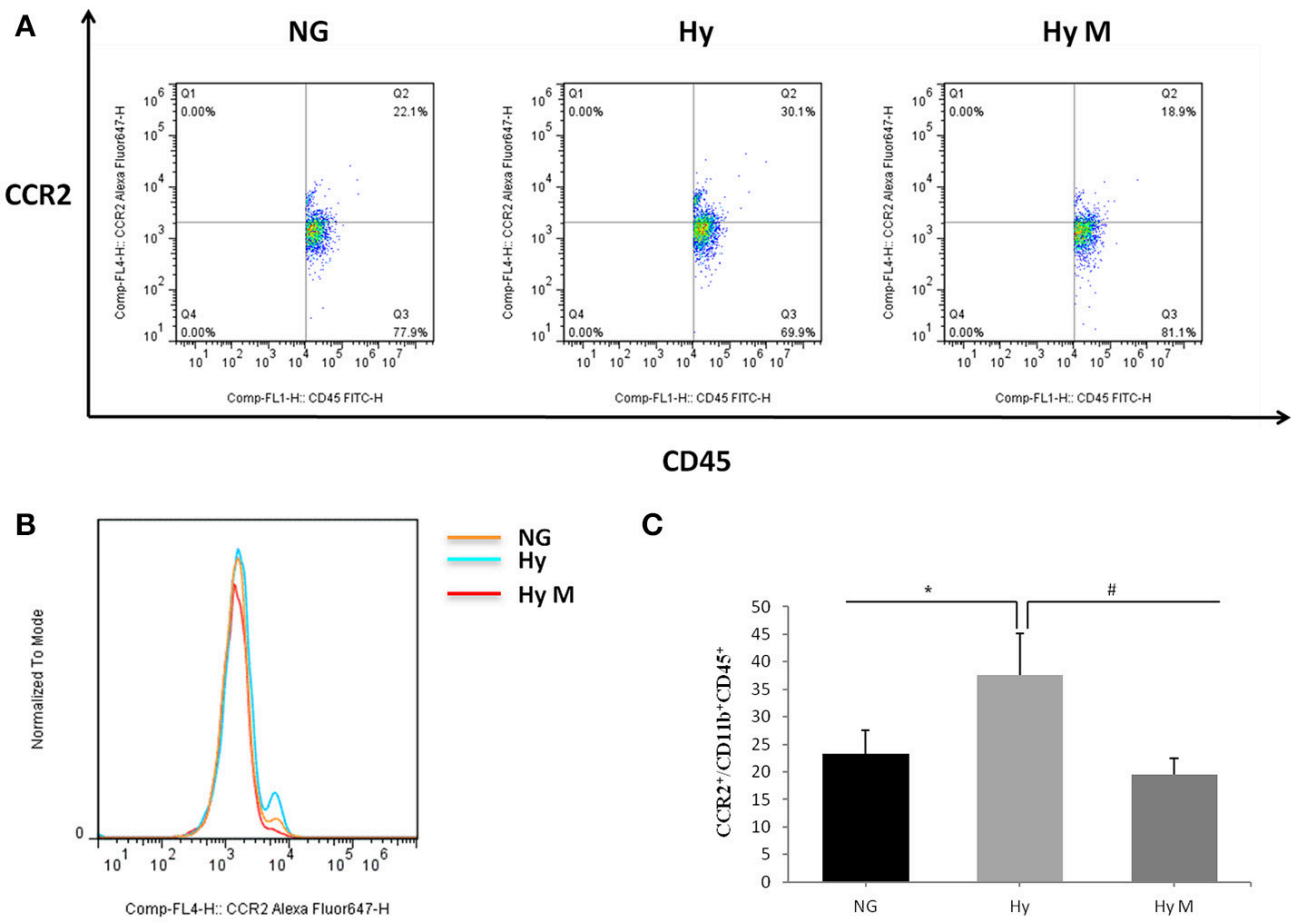
The percentage of CCR2^+^/CD11b^+^CD45^+^ after hypoxia. **(A–C)** Representative percentage **(A)** and histogram **(B)** of CCR2^+^ fluorescence intensity in CD11b^+^CD45^+^ cells after 3.5 h hypoxia and quantitation **(c)** of CCR2^+^/CD11b^+^CD45^+^ after hypoxia in indicated groups are illustrated. ^*^p < 0.05 vs. NG group, ^#^*p* < 0.05 vs. Hy group. *n* = 7 animals per group. Data expressed as means ± SEM. NG represents normal control rats; Hy represents hypoxia rats; Hy M represents hypoxia rats with minocycline treatment.

**Figure 8:**
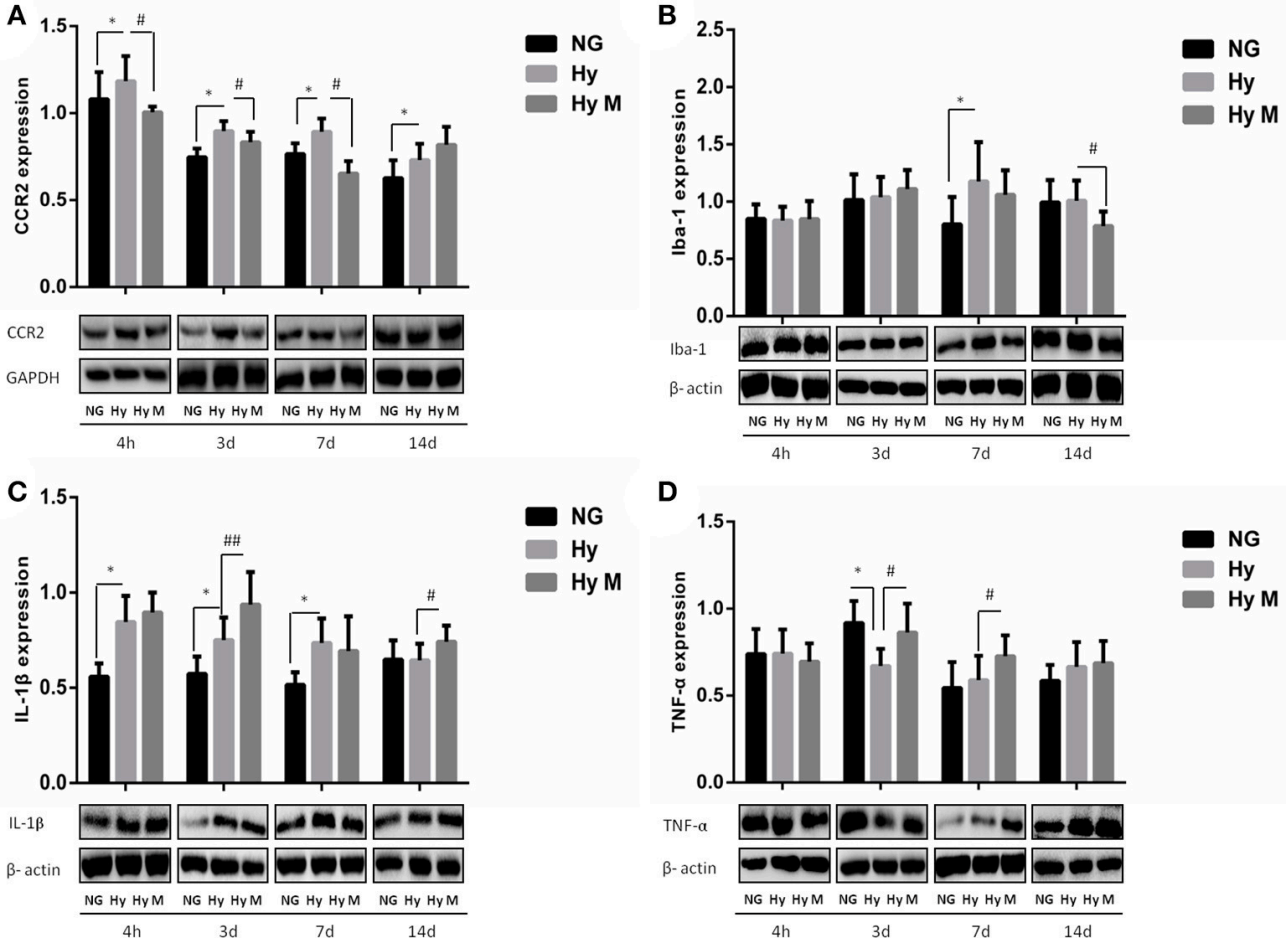
Expression levels of CCR2, Iba-1, IL-1β and TNF-α and in the periventricular zone after 3.5 h hypoxia. **(A)** The representative images and quantification of CCR2 expression that was normalized to GAPDH at indicated time points after hypoxia for 3.5 h were appeared. **(B)** The representative images and quantification of Iba-1 expression that was normalized to β-actin at indicated time points after 3.5 h hypoxia exposure were shown. **(C)** The representative images and quantification of IL-1β that was normalized to β-actin expression at indicated time points after hypoxia (3.5 h) were illustrated. **(D)** The representative images and quantification of TNF-α expression (normalized to β-actin) at indicated time points after hypoxia for 3.5 h were indicated. ^*^p < 0.05 vs. NG group. ^#^*p* < 0.05 and ^##^*p* < 0.01 vs. Hy group. *n* = 5–8 animals per group. Data expressed as means ± SEM. NG represents normal control rats; Hy represents hypoxia rats; Hy M represents hypoxia rats with minocycline treatment.

To test if the increased total number of leukocytes in blood would promote the number of monocytes in the CNS, we performed flow cytometry and immunoblotting assays from the perfused brain tissues. Flow cytometry result indicated that the percentage of CCR2^+^/CD11b^+^CD45^+^ positive cells was significantly increased 3d after hypoxia, which was decreased by minocycline pretreatment (Figures 7A–C; *p* < 0.05 Hy vs. NG, *p* < 0.05 Hy vs. Hy M). Given that CD11b is expressed by microglia and CD45/CCR2 are expressed within peripheral monocytes (Prinz and Priller, 2010), we believe the infiltration of monocytes was obvious. In addition, using immunoblotting we found that CCR2 was significantly increased 4 h after hypoxia, and remained at higher levels for 2 weeks (Figure 8A; *p* < 0.05 Hy vs. NG). While CCR2 can be expressed by microglia as well, the level of Iba-1, an activated microglial cell maker, was not altered until 3 days after hypoxia (Figure 8B; *p* < 0.05 Hy vs. NG). These data suggested that it might exist an initial infiltration of monocytes into the CNS during the first 3d after hypoxia in the P0d rat pups.

We next evaluated microglial activation by measuring the level of inflammatory cytokine IL-1β and TNF-α in the periventricular zone of brain tissue. As shown in Figure 8C, IL-1β was significantly increased from 4 h to 7 days after hypoxia (Figure 8C; *p* < 0.05 Hy vs. NG). But the expression levels of TNF-α declined significantly at 3 days after hypoxia exposure when compared with the corresponding control rats (Figure 8D; *p* < 0.05 Hy vs. NG). Most interestingly, we found that minocycline administration reduced CCR2^+^/CD11b^+^CD45^+^ level at 3d after systemic hypoxia and prevented CCR2 expression (4 h, 3d, 7d after hypoxia), restored Iba-1 expression at 14 days after hypoxia and progressively up-regulate TNF-α expression at 3 days and 7 days after the hypoxia exposure, without inhibiting IL-1β expression at most of the testing time points (4 h, 3d, 7d and 14 d after hypoxia; Figures 8A–D; *p* < 0.05 and *p* < 0.01 Hy vs. Hy M).

**Figure 9:**
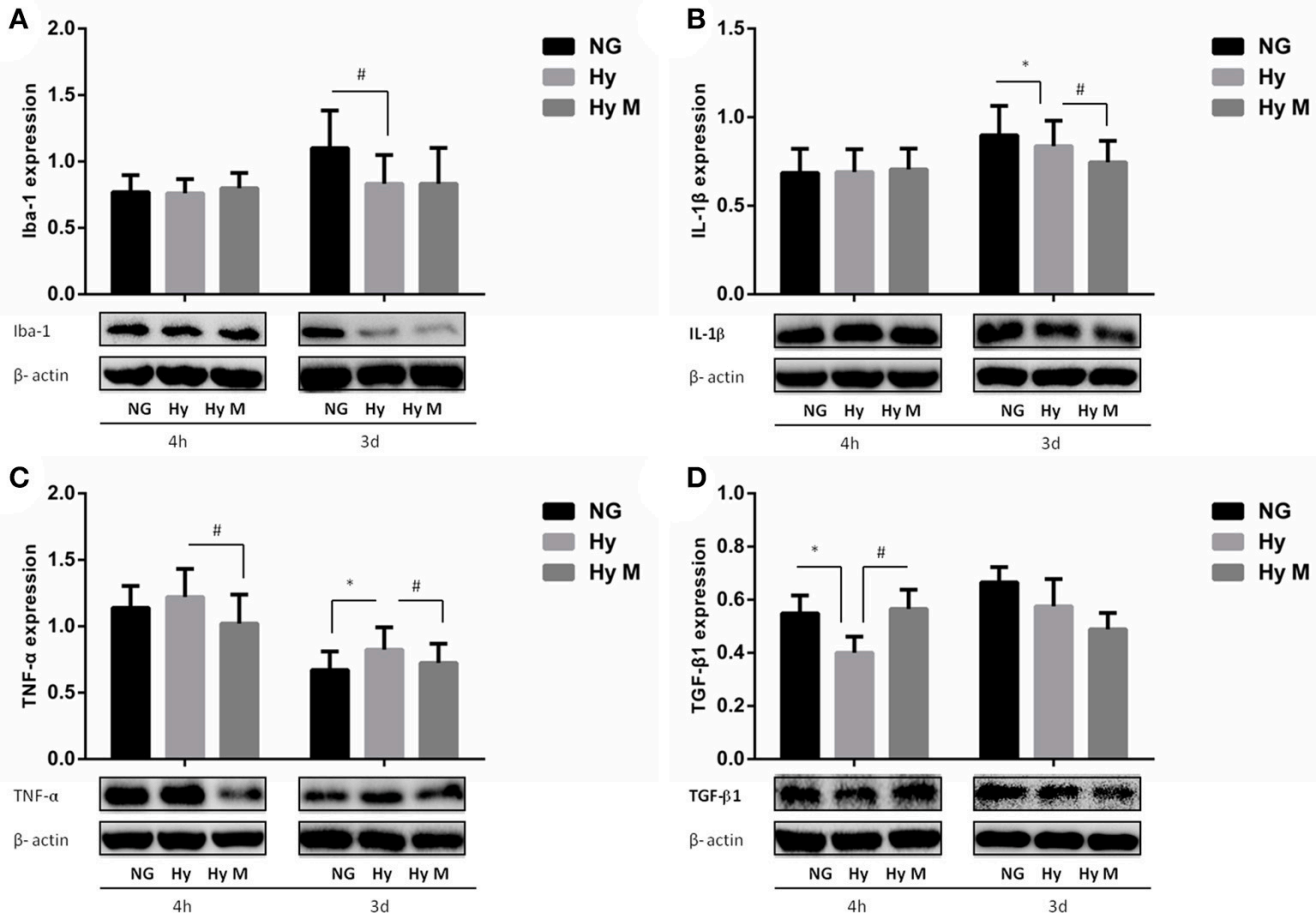
Effect of minocycline on Iba-1, IL-1β, TNF-α, and TGF-β1 in the Hippocampus after 3.5 h hypoxia. **(A)** The representative images and quantification of Iba-1expression that was normalized to β-actin at indicated time points after 3.5 h hypoxia exposure were shown. **(B)** The representative images and quantification of IL-1β that was normalized to β-actin expression at indicated time points after hypoxia (3.5 h) were illustrated. **(C)** The representative images and quantification of TNF-α expression (normalized to β-actin) at indicated time points after hypoxia for 3.5 h were indicated. **(D)** The representative images and quantification of TGF-β1 expression that was normalized to β-actin at indicated time points after hypoxia for 3.5 h were appeared. ^*^p < 0.05 vs. NG group. ^#^*p* < 0.05 vs. Hy group. *n* = 7–9 animals per group. Data expressed as means ± SEM. NG represents normal control rats; Hy represents hypoxia rats; Hy M represents hypoxia rats with minocycline treatment.

In addition, we also investigated the hippocampus inflammation response after hypoxia exposure. Immunoreactive bands of Iba-1, IL-1β, TNF-α, and TGF-β1 protein were measured using western blotting. Iba-1 protein level was significantly decreased compared to the corresponding normal animal at 3 days after hypoxia exposure (Figure 9A; *p* < 0.05 Hy vs. NG), and minocycline administration had not effect on Iba-1 protein expression after neonatal hypoxia (Figure 9A; *p* > 0.05 Hy vs. Hy M). IL-1β was downregulated by hypoxia 3 days after exposure (Figure 9B; *p* < 0.05 Hy vs. NG), which was suppressed by minocycline treatment (Figure 9B; *p* < 0.05 Hy vs. Hy M). The levels of TNF-α were elevated 3 days after hypoxia exposure (Figure 9C; *p* < 0.05 Hy vs. NG), and minocycline administration decreased TNF-α levels at 4 h and 3 d after hypoxia exposure (Figure 9C; *p* < 0.05 Hy vs. Hy M). Additionally, anti-inflammatory cytokine TGF-β1 was decreased 4 h after the neonatal hypoxia (Figure 9D; *p* < 0.05 Hy vs. NG), which was restored by minocycline administration (Figure 9D; *p* < 0.05 Hy vs. Hy M).

## Discussion

In the present study, a significant cognitive deficit and hypomyelination were found in adults after a systemic hypoxia exposure at neonatal development stage. We revealed that circulatory leukocytes and cytokine consistent peripheral inflammation responded to hypoxia insult, and circulatory leukocytes were associated with the effects of hypoxia/minocycline on cognitive functions. Increased leukocytes in blood might increase monocytes (CCR2^+^/CD11b^+^CD45^+^) in the CNS via BBB disruption, which might contribute to the brain damage after systemic hypoxia. While further study is required to elucidate the detailed mechanisms, our data indicate that suppressing the circulatory inflammation response after neonatal hypoxia injury may be beneficial for HIE treatment.

Modern medical care has greatly improved the survival of hypoxia and HIE infants, but neurological impairment is still a frequent burden in survivors. Cognitive deficit has been reported in the Rice-Vannucci neonatal hypoxia-ischemia model (Khatibi et al., 2011; Lin et al., 2014), perinatal asphyxia model (Herrera-Marschitz et al., 2014) and lipopolysaccharide (LPS) induced inflammation combined with HI model (Alexandre et al., 2013). While the systemic hypoxia model has more clinical relevance in clinical HIE (Salmaso et al., 2014), the neurological change is poorly understood. We reported in the present study that learning/memory was afflicted after hypoxia, examined by Y-shape electric maze and Morris water maze, After the systemic hypoxia exposure we can find the animal spend more time in target square and takes longer time/swim longer distance to find the platform. As the swimming distance data we also can understand it's not because of the locomotors problem but the cognitive injury to result it. Cognitive impairment can be recovered partly by minocycline treatment after hypoxia.

Hypomyelination is considered a major pathological change of HIE and has been reported to be associated with cognitive deficits (Wang et al., 2008; Salmaso et al., 2014). Hypomyelination has been found in several HIE models (Fatemi et al., 2011; Volpe et al., 2011); here we examined P60d brains using 7-T MRI/DTI scanning, and our data revealed that myelin sheath direction was reduced by the neonatal systemic hypoxia exposure. An electronic microscope assay also detected the destroyed myelin structure in the brain (data not shown). In this study, we also performed MBP immunostaining and Luxol-fast-blue staining on brains 60 days after hypoxia. Our result indicated a distorted myelin sheath and a thinner corpus callosum after hypoxia exposure. In addition, we observed a protective effect of minocycline against the hypoxia-induced myelin sheath abnormalities. These results suggested that HIE-increased brain inflammation damaged oligodendrocytes, which MBP protein failed to form a consistent direction of myelination.

While the mechanisms of long-term cognitive deficits/hypomyelination in HIE are not fully understood, the brain endogenous inflammation response may play a key role in the pathological changes of HIE-affected brains (Li et al., 2011; Deng et al., 2014; Graf et al., 2014; Kaur et al., 2014). Brain inflammation includes endogenously activated microglia (M1 and M2) and infiltrated leukocytes (monocytes, neutrophils and macrophages) from the circulation into the brain (Arnoux et al., 2013; Oleg et al., 2014). These two types of inflammation responses could contribute to brain injury differently (Kilic et al., 2008; Noël et al., 2012; Arnoux et al., 2013) and remains to be elucidated (Paolicelli et al., 2011). However, it is very hard to technically separate one from the other precisely. Now it becomes clear that microglia and monocytes present different inflammation response and their own cytokine products may play different roles in stroke (Lambertsen et al., 2009). In our study we utilized the CCR2 and CD45 (expressed within peripheral monocytes) and microglia cell marker, CD11b to illustrate the inflammation in the brain after systemic hypoxia. We reported that microglial cell activation and cytokine overexpression happened at different times after hypoxia. Residential microglial cell activation was found at 7 days after hypoxic exposure. However, IL-1β was elevated 4 h and 3 days after hypoxia, while the residential microglial cells remained unchanged. In addition, peripheral total leukocyte number, cytokine expression, and monocytes in brain were increased in the brain. Taken together, we suggested that peripheral leukocytes especially the peripheral monocytes might be involved in IL-1β expression at 4 h and 3 days time points. While we were not able to differentiate the role of leukocyte-originated IL-1β from the role of microglial cell-originated IL-1β in brain damage after hypoxia, our results indicated that the peripheral inflammation response especially leukocyte number and the monocytes in the brain were associated with cognitive dysfunction after systemic hypoxia. Importantly, BBB interruption after hypoxia might provide a way for the monocyte infiltration.

Leukocyte level predicts long-term outcome and hypoxia severity in neonatal hypoxic injury. As peripheral immune cells, leukocytes take part in the peripheral inflammatory response and invade into the CNS after injury (Prinz and Priller, 2010). Given that peripheral leukocytes can be conventionally examined in the clinic setting, some studies attempted to develop leukocytes as a biomarker for brain injury (Morkos et al., 2007; Troger et al., 2013). However, the findings remain controversial. A well-used treatment in clinics for HIE is hypothermia, which results in significantly lower levels of blood leukocytes and its subclasses such as, monocytes, suggesting that targeting leukocytes might be beneficial for neonatal hypoxia-induced brain injury (Jenkins et al., 2013). In order to evaluate the correlation between leukocytes and long-term outcome in our study, we observed a significant correlation between hypoxic exposure time and the peripheral leukocyte number. In addition, leukocyte number and RLCDs were statistically significantly correlated among NG, Hy, and Hy M groups. Our results supported that leukocytes not only responded to hypoxia but also positively correlated with long-term brain function alterations. Therefore, it might be a potential parameter to indicate hypoxic severity and brain injury outcome in neonate hypoxia.

Minocycline preserved cognitive function may involves Leukocytes. Many studies demonstrated minocycline-suppressed myelination degeneration and inflammation in HIE via suppressing microglial cell activation (Fox et al., 2005; Wixey et al., 2011b; Schmitz et al., 2014; Zhu et al., 2014). An obviously inhibition of inflammatory response also be approved in this study. This inhibition by minocycline is detected in the hippocampus only but not the PWM in this systemic hypoxia model. This difference may come from the diverse function of microglial cell in different region in the brain, which remains further study. However, minocycline's effects on circulation inflammation are not clear. Consistent with previous findings, our data demonstrated that minocycline reversed neurological impairment and hypomyelination. Most significantly, we also indicated that minocycline inhibited circulatory inflammatory response especially leukocyte and invading monocyte activation as well, without changing microglial cell activation in PWM but suppress inflammation in the hippocampus at the early time points after systemic hypoxia. Our findings is supportive to a previous study, in which minocycline inhibited the IL-1β and TNFα expression in the circulation but only had a weak effect on the brain after a transient middle cerebral artery occlusion to neonatal mice (Fox et al., 2005).

## Conclusion

Systemic hypoxia induced cognitive deficits and hypomyelination. Peripheral inflammation is involved in neuroinflammation and circulatory leukocyte activation may become a potential parameter to evaluate hypoxia severity and outcome. The beneficial effect of minocycline on systemic hypoxia-induced brain damage involves its role in leukocyte activation and cytokine release in the peripheral blood. Thus, increasing the number of anti-leukocyte cells or suppressing circulatory inflammation after neonatal hypoxia may be a promising strategy for the recovery of hypoxia-affected newborns.

## Author contributions

YM performed flow cytometry, LFB staining, and immunofluorescence /immunochemistry staining for MBP, HL performed Elisa analysis, Iba-1\TNF-α\IL-1β western blotting assays, KX, WW, and YH performed leukocyte number count. YH, LL, TY, LH, LY, and HJ, performed Y-maze behavior experiment. YH, LL, TY, and LH, performed Morris water maze behavior test. JX performed the 7-TMRI scanning and FA value. JX, KX, HL, and YM performed statics analysis. FL wrote the main manuscript text and YM helped finish the manuscript text, HL, KX, and YM prepared figures. All authors reviewed the manuscript.

### Conflict of interest statement

The authors declare that the research was conducted in the absence of any commercial or financial relationships that could be construed as a potential conflict of interest.

## References

[B1] Alexandre S. Karine L. Marie E. B. Djordje G. Martin L. Denis G. (2013). Involvement of neuronal IL-1β in acquired brain lesions in a rat model of neonatal encephalopathy. J. Neuroinflammation 10:110 10.1186/1742-2094-10-110 24007297 PMC3844447

[B2] Anthony D. C. Couch Y. Losey P. Evans M. C. (2012). The systemic response to brain injury and disease. Brain Behav. Immun. 26, 534–540. 10.1016/j.bbi.2011.10.011 22085588

[B3] Arnoux I. Hoshiko M. Mandavy L. Avignone E. Yamamoto N. Audinat E.. (2013). Adaptive phenotype of microglial cells during the normal postnatal development of the somatosensory “Barrel” cortex. Glia 61, 1582–1594. 10.1002/glia.22503 23893820

[B4] Back S. A. Luo N. L. Borenstein N. S. Levine J. M. Volpe J. J. Kinney H. C. (2001). Late oligodendrocyte progenitors coincide with the developmental window of vulnerability for human perinatal white matter injury. J. Neurosci. 21, 1302–1312. 11160401 10.1523/JNEUROSCI.21-04-01302.2001 PMC6762224

[B5] Bonestroo H. J. C. Nijboer C. H. A. van Velthoven C. J. Kavelaars A. Hack C. E. van Bel F.. (2013). Cerebral and hepatic inflammatory response after neonatal hypoxia-ischemia in newborn rats. Dev. Neurosci. 35, 197–211. 10.1159/000346685 23689428

[B6] Brundula V. Rewcastle N. B. Metz L. M. Bernard C. C. Yong V. W. (2002). Targeting leukocyte MMPs and transmigration Minocycline as a potential therapy for multiple sclerosis. Brain 125:11. 10.1093/brain/awf133 12023318

[B7] Deng Y. Y. Xie D. Fang M. Zhu G. F. Chen C. B. Zeng H. K.. (2014). Astrocyte-derived proinflammatory cytokines induce hypomyelination in the periventricular white matter in the hypoxic neonatal brain. PLoS ONE 9:e87420. 10.1371/journal.pone.0087420 24498101 PMC3909103

[B8] Fatemi A. Wilson M. A. Phillips A. W. McMahon M. T. Zhang J. Y. Smith S. A.. (2011). *In vivo* magnetization transfer MRI shows dysmyelination in an ischemic mouse model of periventricular leukomalacia. J. Cereb. Blood Flow Metab. 31, 2009–2018. 10.1038/jcbfm.2011.68 21540870 PMC3208153

[B9] Fox C. Dingman A. Derugin N. Wendland M. F. Manabat C. Ji S.. (2005). Minocycline confers early but transient protection in the immature brain following focal cerebral ischemia-reperfusion. J. Cereb. Blood Flow Metab. 25, 1138–1149. 10.1038/sj.jcbfm.9600121 15874975 PMC2262097

[B10] Graf A. E. Haines K. M. Pierson C. R. Bolon B. N. Houston R. H. Velten M.. (2014). Perinatal inflammation results in decreased oligodendrocyte numbers in adulthood. Life Sci. 94, 164–171. 10.1016/j.lfs.2013.11.015 24291255 PMC3923532

[B11] Herrera-Marschitz M. Neira-Pena T. Rojas-Mancilla E. Espina-Marchant P. Esmar D. Perez R.. (2014). Perinatal asphyxia: CNS development and deficits with delayed onset. Front. Neurosci. 8:47. 10.3389/fnins.2014.00047 24723845 PMC3972459

[B12] Jenkins D. D. Lee T. Chiuzan C. Perkel J. K. Rollins L. G. Wagner C. L.. (2013). Altered circulating leukocytes and their chemokines in a clinical trial of therapeutic hypothermia for neonatal hypoxic ischemic encephalopathy. Pediatr. Crit. Care Med. 14, 786–795. 10.1097/PCC.0b013e3182975cc9 23897243

[B13] Kaur C. Sivakumar V. Zou Z. Ling E.-A. (2014). Microglia-derived proinflammatory cytokines tumor necrosis factor-alpha and interleukin-1beta induce Purkinje neuronal apoptosis via their receptors in hypoxic neonatal rat brain. Brain Struct. Funct. 219, 151–170. 10.1007/s00429-012-0491-5 23262920

[B14] Khan M. B. Hoda M. N. Vaibhav K. Giri S. Wang P. Waller J. L.. (2015). Remote ischemic postconditioning: harnessing endogenous protection in a murine model of vascular cognitive impairment. Transl. Stroke Res. 6, 69–77. 10.1007/s12975-014-0374-6 25351177 PMC4297613

[B15] Khatibi N. H. Lee L. K. Zhou Y. L. Chen W. Q. Rolland W. Fathali N.. (2011). Endothelin receptor-A (ETa) inhibition fails to improve neonatal hypoxic-ischemic brain injury in rats. Acta Neurochir. Suppl. 111, 207–212. 10.1007/978-3-7091-0693-8_35 21725757

[B16] Kichev A. Rousset C. I. Baburamani A. A. Levison S. W. Wood T. L. Gressens P.. (2014). Tumor necrosis factor-related apoptosis-inducing ligand (TRAIL) signaling and cell death in the immature central nervous system after hypoxia-ischemia and inflammation. J. Biol. Chem. 289, 9430–9439. 10.1074/jbc.M113.512350 24509861 PMC3979382

[B17] Kilic U. Kilic E. Matter C. M. Bassetti C. L. Hermanna D. M. (2008). TLR-4 deficiency protects against focal cerebral ischemia and axotomy-induced neurodegeneration. Neurobiol. Dis. 31, 33–40. 10.1016/j.nbd.2008.03.002 18486483

[B18] Lambertsen K. L. Clausen B. H. Babcock A. A. Gregersen R. Fenger C. Nielsen H. H.. (2009). Microglia protect neurons against ischemia by synthesis of tumor necrosis factor. J. Neurosci. 29, 1319–1330. 10.1523/JNEUROSCI.5505-08.2009 19193879 PMC6666095

[B19] Li F. Faustino J. Woo M. S. Derugin N. Vexler Z. S. (2015). Lack of the scavenger receptor CD36 alters microglial phenotypes after neonatal stroke. J. Neurochem. 135, 445–452. 10.1111/jnc.13239 26223273 PMC4844456

[B20] Li F. Wang L. Li J. W. Gong M. He L. Feng R.. (2011). Hypoxia induced amoeboid microglial cell activation in postnatal rat brain is mediated by ATP receptor P2X4. BMC Neurosci. 12:111. 10.1186/1471-2202-12-111 22053919 PMC3239293

[B21] Lin E. P. Miles L. Hughes E. A. McCann J. C. Vorhees C. V. McAuliffe J. J.. (2014). A combination of mild hypothermia and sevoflurane affords long-term protection in a modified neonatal mouse model of cerebral hypoxia-ischemia. Anesth. Analg. 119, 1158–1173. 10.1213/ANE.0000000000000262 24878681

[B22] Meng S. Qiao M. Scobie K. Tomanek B. Tuor U. I. (2006). Evolution of magnetic resonance imaging changes associated with cerebral hypoxia-ischemia and a relatively selective white matter injury in neonatal rats. Pediatr. Res. 59, 554–559. 10.1203/01.pdr.0000203099.40643.84 16549528

[B23] Morkos A. A. Hopper A. O. Deming D. D. Yellon S. M. Wycliffe N. Ashwal S.. (2007). Elevated total peripheral leukocyte count may identify risk for neurological disability in asphyxiated term neonates. J. Perinatol. 27, 365–370. 10.1038/sj.jp.7211750 17443199

[B24] Noël C. D. James C. C. Zhen J. L. Eric X. Stephen B. G. A. Patrice G. G. (2012). Wild-type microglia arrest pathology in a mouse model of rett syndrome. Nature 484, 105–109. 10.1038/nature10907 22425995 PMC3321067

[B25] Oleg B. Mark P. J. Craig S. M. Ron C. Amanda J. L. Galina G. (2014). Identification of a unique TGF-β dependent molecular and functional signature in microglia. Nat. Neurosci. 17, 131–143. 10.1038/nn.3599 24316888 PMC4066672

[B26] Paolicelli R. C. Bolasco G. Pagani F. Maggi L. Scianni M. Panzanelli P.. (2011). Synaptic pruning by microglia is necessary for normal brain development. Science 333, 1456–1458. 10.1126/science.1202529 21778362

[B27] Prinz M. Priller J. (2010). Tickets to the brain: role of CCR2 and CX3CR1 in myeloid cell entry in the CNS. J. Neuroimmunol. 224, 80–84. 10.1016/j.jneuroim.2010.05.015 20554025

[B28] Salmaso N. Jablonska B. Scafidi J. Vaccarino F. M. Gallo V. (2014). Neurobiology of premature brain injury. Nat. Neurosci. 17, 341–346. 10.1038/nn.3604 24569830 PMC4106480

[B29] Schmitz T. Krabbe G. Weikert G. Scheuer T. Matheus F. Wang Y.. (2014). Minocycline protects the immature white matter against hyperoxia. Exp. Neurol. 254, 153–165. 10.1016/j.expneurol.2014.01.017 24491957

[B30] Semple B. D. Blomgren K. Gimlin K. Ferriero D. M. Noble-Haeusslein L. J. (2013). Brain development in rodents and humans: identifying benchmarks of maturation and vulnerability to injury across species. Prog. Neurobiol. 106–107, 1–16. 10.1016/j.pneurobio.2013.04.001 PMC3737272 23583307

[B31] Troger B. Muller T. Faust K. Bendiks M. Bohlmann M. K. Thonnissen S.. (2013). Intrauterine growth restriction and the innate immune system in preterm infants of </=32 weeks gestation. Neonatology 103, 199–204. 10.1159/000343260 23295537

[B32] Vannucci S. J. Hagberg H. (2004). Hypoxia-ischemia in the immature brain. J. Exp. Biol. 207, 3149–3154. 10.1242/jeb.01064 15299036

[B33] Vexler Z. S. Yenari M. A. (2009). Does inflammation after stroke affect the developing brain differently than adult brain? Dev. Neurosci. 31, 378–393. 10.1159/000232556 19672067 PMC2790734

[B34] Volpe O. J. Kinney H. C. Frances E. J. Rosenberg P. A. (2011). The developing oligodendrocyte: key cellular target in brain injury in the premature infant. Int. J. Dev. Neurosci. 29, 423–440. 10.1016/j.ijdevneu.2011.02.012 21382469 PMC3099053

[B35] Vorhees C. V. Williams M. T. (2006). Morris water maze: procedures for assessing spatial and related forms of learning and memory. Nat. Protoc. 1, 848–858. 10.1038/nprot.2006.116 17406317 PMC2895266

[B36] Wang S. L. Wu E. X. Tam C. N. Lau H. F. Cheung P. T. Khong P. L.. (2008). Characterization of white matter injury in a hypoxic-ischemic neonatal rat model by diffusion tensor MRI. Stroke 39, 2348–2353. 10.1161/STROKEAHA.107.509927 18535275

[B37] Wixey J. A. Reinebrant H. E. Buller K. M. (2011a). Inhibition of neuroinflammation prevents injury to the serotonergic network after hypoxia-ischemia in the immature rat brain. J. Neuropathol. Exp. Neurol. 70, 23–35. 10.1097/NEN.0b013e3182020b7b 21157380

[B38] Wixey J. A. Reinebrant H. E. Spencer S. J. Buller K. M. (2011b). Efficacy of post-insult minocycline administration to alter long-term hypoxia-ischemia-induced damage to the serotonergic system in the immature rat brain. Neuroscience 182, 184–192. 10.1016/j.neuroscience.2011.03.033 21440046

[B39] Xu K. Y. Du X. H. Wang W. Z. Ciu X. Li H. J. Zhou F. Y. (2013). Correlation between routine blood indexes and postnatal development and change of serum iron concentration in neonatal SD rats. Chin. J. Pathophysiol. 29, 2256–2262. 10.3969/j.issn.1000-4718.2013.12.023

[B40] Zhu F. R. Zheng Y. J. Ding Y. Q. Liu Y. Zhang X. H. Wu R. R.. (2014). Minocycline and risperidone prevent microglia activation and rescue behavioral deficits induced by neonatal intrahippocampal injection of lipopolysaccharide in rats. PLoS ONE 9:e93966. 10.1371/journal.pone.0093966 24705495 PMC3976322

